# On the Treatment of Bronchocele by Compression

**Published:** 1851-01

**Authors:** Wm. C. Dwight

**Affiliations:** Moscow, N. Y.


ART. III.—*On the Treatment of Bronchocele by Compression.* By Wm. C. Dwight, M. D., of Moscow, N. Y.

Although Goitre is by no means common, yet it is not so rare in some districts of our country as not to require attention.

Many cases were brought under my notice when Iodine had become the fashionable remedy, and my patients were advised in regard to its use. All the precautions were taken to have them guarded from the effects of imprudent use of this medicine, yet, more than once, I was forced to witness distress for breath, and palpitation of the heart, which I could attribute to nothing but the Iodine, and this, too, before there was any sensible diminution of the deformity. It was found, moreover, that this was an evil to be expected, as prudence is not common at the age of patients of this class. Under such circumstances, it was desirable to look about for a safer remedy, and it was determined to try pressure. To produce sufficient pressure without impeding respiration, resort was had to the following mode of proceeding:—

Three straps of good glazed brown cambric were spread with Emp. Ol. Lini cum Plumb. Sem. Vit. Oxidi.,* each of half the width of the tumor, and of length sufficient to reach from the lower edge of the scapula of one side obliquely up the opposite side of the neck and across the lower part of the tumor, passing thence onward in return to the upward direction down to the lower edge of opposite scapula, crossing like suspenders. The strap is drawn quite tightly, producing very considerable turgescence of the blood-vessels of the face. The patient will shrug up his shoulders for a few minutes until the Thyroid vessels become compressed sufficiently to enable him to breathe more comfortably, and the countenance resumes its natura appearance. Five minutes is all the time ordinarily required. The second strap is then passed in the same manner across the upper part, from half an inch, to an inch, from the first, according to the circumstances of the case, such as length of neck, size of tumor or situation and form. This strap is drawn as tightly as the first. After waiting until the countenance allows a new application, the third strap is put on in the same manner over the intermediate space in like fashion.

Ordinarily the plasters will adhere in cool weather from ten days to a fortnight, when, becoming loose and non-adherent, they ought to be removed. If the pressure has been well applied, the tumor will be found to have become slightly less, the skin somewhat reddened and tender. In such case it is prudent to wait until it assumes its natural appearance before a new application of the plasters.

The first application has in one case been sufficient, but the average has been as high as four times in each case. When the Bronchocele has become diminished to half its size at the time of the first application, it will continue to disappear without further care. The success which has attended this treatment is such as to warrant confidence. In twenty cases there has been no failure. In the first four, Iodine was used in conjunction with the plasters, and in the twelfth it was used antecedently for several weeks without diminution of the disease. In these cases the progress was no more rapid than when no Iodine was used. In two of the cases the disease returned at the end of two years each, but on a new application of the straps was immediately overcome, and although ten years have elapsed

---

* I prefer this plaster as I know of no other with equal adhesive property, which produces so little irritation.

since the last application, all is as well yet as though there had never been any deformity. It is proper to add, also, that in both of these cases Iodine was freely taken as well as pressure used at the commencement.

Moscow, Dec. 1850.

